# Pandemic Exposure, Post-traumatic Stress Disorder, Conflict Behaviors, and Online Aggressive Behaviors Among College Students During the COVID-19 Pandemic: Examining the Moderating Role of Gender

**DOI:** 10.3389/fpsyt.2022.809173

**Published:** 2022-03-17

**Authors:** Baohua Zhen, Benxian Yao, Xiao Zhou

**Affiliations:** ^1^College of Educational Science, Anhui Normal University, Wuhu, China; ^2^Department of Psychology and Behavioral Sciences, Zhejiang University, Hangzhou, China

**Keywords:** pandemic exposure, PTSD, conflict behaviors, online aggressive behaviors, gender

## Abstract

The COVID-19 pandemic impacts people’s mental health and behaviors, but the influence mechanism between pandemic exposure, conflict behaviors, and online aggressive behaviors during the COVID-19 outbreak remains unclear. This study will address this gap. Data were collected from 1,153 college students in an online survey that included an pandemic experiences scale, a post-traumatic stress disorder (PTSD) scale, a conflict behaviors scale, and an online aggressive behaviors scale. Structural equation modeling and multi-group comparisons were used to analyze the data. Pandemic exposure positively predicted conflict and online aggressive behaviors through hyperarousal symptoms, and negatively predicted these behaviors through intrusive symptoms. The mediating roles of avoidance and negative alterations in cognition and mood symptoms in the relations between pandemic exposure and conflict behaviors and online aggressive behaviors were non-significant. Among male students, pandemic exposure directly predicted conflict and online aggressive behaviors, but for female students, pandemic exposure indirectly influenced these behaviors through intrusive and hyperarousal symptoms. The intrusive and hyperarousal symptoms play mediating roles in the relations between pandemic exposure and conflict behaviors and online aggressive behaviors. Gender plays a moderating role in the above mediating mechanism.

## Introduction

The Coronavirus disease 2019 (COVID-19) pandemic is considered as the most crucial global health calamity of the century (1). The pandemic not only caused tremendous economic losses to society, but also negatively impacted people’s physical and mental health. For example, since the COVID-19 outbreak, many accusations were made over the Internet. Some people abused the COVID-19 patients online, adopted hostile attitudes toward residents in pandemic areas and accused them of spreading the virus. These accusations even escalated into regional attacks, cyber manhunt, and other online aggressive behaviors that caused huge psychological pressure for the public (2). There were also some real-world conflict behaviors that disturbed the social living order and affected social stability. People’s daily lives, mental health, and physical health were affected by these conflict behaviors and online aggressive behaviors.

Previous studies have suggested that traumatized individuals may show increased aggressive behaviors (3, 4). Researchers have also found that during the COVID-19 pandemic in early 2020, conflicts and cyberbullying have risen sharply (5). For example, among the young people who experienced COVID-19 pandemic and quarantine, their aggressive behaviors increased significantly (6, 7). As individuals who had severer traumatic exposure tended to experience high levels of anger and fear, they were more likely to have aggressive behaviors (7, 8). In addition, traumatic experiences may reduce individuals’ sense of intimacy and trust in others (9, 10), which makes it difficult for them to get along with others, inducing more interpersonal conflicts and aggression. Therefore, traumatized individuals show more aggressive behaviors (11). Many empirical studies have found a correlation between traumatic experiences and aggressive behaviors (12, 13), but most studies focused on trauma exposure through events such as natural disasters and wars. Few studies have examined the correlation between trauma exposure and aggressive behaviors in the context of a public health emergency. COVID-19 is a typical public health emergency and can be considered as a traumatic event, which may elicit PTSD-like responses and exacerbate other related mental health problems (14, 15). And it is worth noting that home quarantine was an important measure for pandemic prevention and control during the COVID-19 pandemic. During this period, people’s scope of activities was limited, and they spent more time online. Their aggressive behaviors may therefore have manifested not only as conflict behaviors in daily life, but also as online aggressive behaviors in the virtual world. Therefore, this study assessed the impact of exposure to the COVID-19 pandemic on conflict behaviors and online aggressive behaviors.

Post-traumatic stress disorder (PTSD) may also play an important role in the correlation between pandemic exposure and conflict behaviors and online aggressive behaviors. Studies have shown that individuals who had experienced major disaster (e.g., an earthquake, war) reported a variety of psychological problems. PTSD is one of the most common problems in this context (16–19). PTSD symptoms of varying degrees were found in 12.8% of youth people who had exposure to the COVID-19 pandemic in China (20). The detection rate of PTSD symptoms among college students was estimated at 30.8% (21). In addition, an individual’s PTSD symptoms are closely related to their aggressive behaviors (22, 23). Compared with psychiatrically healthy ones, individuals with PTSD showed a stronger tendency toward aggression (24, 25). The cognitive action theory of PTSD suggests that PTSD causes cognitive bias in the processing of threatening information; therefore, mild evidence of threat activates threat-response structures that bias the individual to interpret ambiguous evidence as threatening. Their alertness to threats is increased, thereby affecting their cognition and emotion, and leading to more aggressive behaviors (8, 26). In addition, individuals with PTSD may expect to reduce their negative emotions by engaging in aggressive behaviors (27, 28). It is worth noting that PTSD contains four symptom clusters [intrusive symptoms, avoidance symptoms, negative alterations in cognition and mood (NACM) symptoms, and hyperarousal symptoms], each of which has different manifestations (29). Thus, it is possible that these four symptom clusters exert different impact on individuals’ aggressive behaviors. Therefore, this study assumed that PTSD symptom clusters mediated the correlation between pandemic exposure and conflict behaviors and online aggressive behaviors (hypothesis 1).

Gender may also play a moderating role in the relations between pandemic exposure and conflict behaviors and online aggressive behaviors. A previous study found that the positive rate of PTSD in females was significantly higher than that in males (30). Other studies have shown that males were more aggressive than females after traumatic events (31); males tended to be more aggressive, whereas females reported higher PTSD symptoms (32). In addition, females showed more emotional problems (33) and males showed more aggressive behaviors (23). These findings suggest that the effects of pandemic exposure on PTSD and aggressive behaviors differ between males and females. Augsburger and Maercker (34) reported that among individuals with PTSD, males and females also had different performance. For example, males scored higher than females on aggression, anger, and verbal hostility scales (35, 36). These findings suggest that there are gender differences in the relation between PTSD and aggressive behaviors. Therefore, this study assumed that gender plays a moderating role in the correlations between pandemic exposure, PTSD, conflict behaviors, and online aggressive behaviors (hypothesis 2).

In China, college students come from different places around the country, especially given the enormous scale of population migration. Therefore, all Chinese colleges postponed school semesters in response to the pandemic prevention and control requirements, which means college students’ free time was extended. At present, college students belong to the cohort of millennials and digital natives. Not only do they receive a lot of information from the Internet in their free time, but it also plays an important role in network communication (37). These students were one of the representative groups affected by COVID-19. Therefore, this study investigated college students and compared the mediating role of PTSD symptom clusters in the relations between pandemic exposure and conflict behaviors and online aggressive behaviors in male and female students.

## Materials and Methods

### Participants and Procedure

The investigation was conducted during May 9th to May 20th, 2020, about 4 months after the outbreak of COVID-19 pandemic. At this time, most schools remained closed to prevent and control the pandemic, and college students kept learning online at home, so this investigation was carried out by Wenjuanxing (an online survey platform). The online questionnaires were spread via WeChat (a free messaging and calling app commonly used in China) directly to college students, and we also asked college teachers or related workers to forward the questionnaires. A total of 1,325 questionnaires was gathered, as all of the items in the questionnaires were choice-forced, there was no missing data. In order to ensure the quality of questionnaires, students who submitted the questionnaire within 2 min and those who answered randomly or regularly were excluded, and 1,153 questionnaires were finally retained.

Of these participants, 602 (52.2%) participants were female and 551 (47.8%) were male, 665 (57.7%) came from the countryside and 488 (42.3%) lived in the city. Participants’ age ranged from 17 to 25 years, with a mean age of 20.2 ± 1.38 years. This study was approved by the Research Ethics Committee of the Department of Psychology and Behavioral Sciences, Zhejiang University. Informed consent was obtained from all participants and no compensation was provided to participants.

### Measures

#### Pandemic Exposure

We used the pandemic experiences scale developed by Zhen and Zhou (38) to assess the degree of pandemic exposure among college students. This scale contains 10 items (e.g., “I became infected during COVID-19 outbreak,” “I was quarantined during COVID-19 outbreak”), each of which has “yes” and “no” response options (no = 1 and yes = 2). The reliability of the scale was acceptable in this study (Cronbach’s alpha = 0.68).

#### Post-traumatic Stress Disorder Symptoms

In this study, the PTSD Checklist from the Diagnostic and Statistical Manual of Mental Disorders, Fifth Edition was used to investigate PTSD symptoms (39). This scale evaluates PTSD symptoms in the past 2 weeks, and has four subscales: intrusive symptoms, avoidance symptoms, NACM symptoms, and hyperarousal symptoms. The entire scale has 20 items, and each item is rated on a 5-point Likert-type scale from 0 to 4 (0 = not at all/only once and 4 = almost every day). In this study, the internal consistency reliability of the scale was good (Cronbach’s alpha = 0.96).

#### Conflict Behaviors

We used a self-developed conflict behaviors scale to assess participants’ conflict behaviors. This scale was developed by revising the Chinese version of the Buss-Perry Aggression Questionnaire in Adolescents (40). The conflict behaviors scale has four items (e.g., “I had physical conflicts with others”), and each item is rated on a 3-point Likert-type scale from 1 to 3 (1 = no and 3 = always). The reliability of the conflict behaviors scale was good in this study (Cronbach’s alpha = 0.75).

#### Online Aggressive Behaviors

We adopted and revised the overt aggression dimension of the Instrumental Online Aggression Scale (41) to evaluate participants’ online aggressive behaviors. The original dimension has seven items, and we deleted three items as these items are not in line with the actual life of today’s college students (e.g., the “MSN” and “Feixin” described in one item are two outdated applications years ago). This revised scale has four items (e.g., “During the pandemic, I have made malicious or hurtful comments on someone on the Internet”), with each item rated on a 3-point Likert-type scale from 1 to 3 (1 = no, 3 = always). In this study, the internal consistency reliability of the online aggressive behaviors scale was good (Cronbach’s alpha = 0.79).

### Data Analysis

We used SPSS 18.0 and Amos 24.0 to conduct descriptive statistical analysis and model analysis, respectively. First, Pearson’s product-moment correlation was used to examine the correlation between major variables. Then, a direct effects model with paths from pandemic exposure to conflict behaviors and online aggressive behaviors was established. Based on the direct model, we inserted the four PTSD symptom clusters as mediators, and developed a final indirect effects model. Finally, we constrained the non-significant paths to zero, thereby establishing a parsimonious path model. Chi-square values, the comparative fit index (CFI), the Tucker–Lewis index (TLI), and the root mean square error of approximation (RMSEA) were used to evaluate model fit. The critical values of model fit were: CFI > 0.90, TLI > 0.90, and RMSEA < 0.08 (42, 43). The final results showed that the model fit the data well.

## Results

### Correlations Between Gender, Pandemic Exposure, Post-traumatic Stress Disorder Symptom Clusters, Conflict Behaviors, and Online Aggressive Behaviors

Table 1 shows the correlations between gender, pandemic exposure, PTSD symptom clusters, conflict behaviors, and online aggressive behaviors. Gender had significant associations with pandemic exposure, avoidance symptoms, and online aggressive behaviors. Pandemic exposure had positive associations with all variables except for avoidance symptoms. There were significant positive pairwise correlations between intrusive symptoms, avoidance symptoms, NACM symptoms, and hyperarousal symptoms. These four symptom clusters had significant and positive correlations with conflict behaviors and online aggressive behaviors. A significant and positive correlation was also found in the relation between conflict behaviors and online aggressive behaviors.

**TABLE 1:** Descriptive statistics and correlations among main variables.

Variables	*M* (*SD*)	1	2	3	4	5	6	7
1. Gender	–	1						
2. Pandemic exposure	12.43 (1.68)	0.08**	1					
3. Intrusive symptom	10.77 (3.46)	0.05	0.07*	1				
4. Avoidance symptom	4.45 (1.52)	−0.08*	0.01	0.72***	1			
5. NACM symptom	15.70 (4.92)	−0.03	0.06*	0.79***	0.75***	1		
6. Hyperarousal symptom	13.80 (4.51)	0.01	0.07*	0.80***	0.69***	0.88***	1	
7. Conflict behaviors	5.02 (1.40)	0.04	0.11**	0.19***	0.18***	0.29***	0.34***	1
8. Online aggressive behaviors	4.64 (1.28)	−0.22***	0.10**	0.10**	0.14**	0.17***	0.18***	0.35***

***p < 0.001, **p < 0.01, *p < 0.05. NACM, negative alterations in cognition and mood; M, mean; SD, standard deviation.

### Testing the Mediating Roles of Post-traumatic Stress Disorder Symptom Clusters

To examine the mediating roles of PTSD symptom clusters in the relations between pandemic exposure and conflict behaviors and online aggressive behaviors, we established a direct effects model with paths from pandemic exposure to conflict behaviors and online aggressive behaviors. Because of the high correlation between conflict behaviors and online aggressive behaviors, we established a correlation path between the two dependent variables to avoid type I error. The model fit the data completely: χ^2^(0) = 0 and CFI = 1.00. The path analysis revealed that pandemic exposure directly predicted both conflict behaviors (β = 0.112, *p* < 0.001) and online aggressive behaviors (β = 0.101, *p* < 0.001).

Based on the direct effects model, we inserted the four PTSD symptom clusters as mediators between pandemic exposure and conflict behaviors and online aggressive behaviors. Then, we established pairwise correlations between the four symptom clusters, and developed a final indirect effects model. The final indirect effects model fit the data completely: χ^2^(0) = 0 and CFI = 1.00. Path analysis revealed five non-significant predictive paths: from pandemic exposure to avoidance symptoms (β = 0.011, *p* > 0.05); from avoidance symptoms to conflict behaviors (β = –0.059, *p* > 0.05) to online aggressive behaviors (β = 0.087, *p* > 0.05); and from NACM symptoms to conflict behaviors (β = 0.095, *p* > 0.05) to online aggressive behaviors (β = 0.074, *p* > 0.05); the other predictive paths were significant.

**FIGURE 1:**
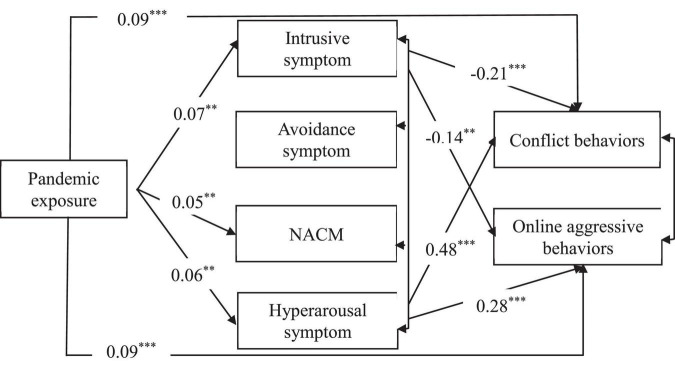
The parsimonious path model of PTSD. ***p < 0.001, **p < 0.01, NACM, negative alterations in cognition and mood.

Next, we constrained these non-significant paths to zero and established a parsimonious path model that fit the data well (see Figure 1): χ^2^(5) = 13.901, CFI = 0.998, TLI = 0.991, and RMSEA = 0.039. The path analysis showed that pandemic exposure positively predicted conflict behaviors (β = 0.09, *p* < 0.001), online aggressive behaviors (β = 0.09, *p* < 0.001), intrusive symptoms (β = 0.07, *p* < 0.01), NACM symptoms (β = 0.05, *p* < 0.01), and hyperarousal symptoms (β = 0.06, *p* < 0.01). Intrusive symptoms negatively predicted conflict behaviors (β = –0.21, *p* < 0.001) and online aggressive behaviors (β = –0.14, *p* < 0.01), and hyperarousal symptoms positively predicted conflict behaviors (β = 0.48, *p* < 0.001) and online aggressive behaviors (β = 0.28, *p* < 0.001). In short, these results showed that pandemic exposure negatively predicted conflict behaviors and online aggressive behaviors through intrusive symptoms, and positively predicted these behaviors via hyperarousal symptoms.

### Testing the Moderating Role of Gender

Structural equation modeling and multi-group comparisons were used to investigate the differences between male and female participants (Figure 1). We defined two models with nested relations: Model 1 (zero model) was defined as the male and female groups having the same model structure, and there were no restrictions on the parameters in the model. Based on Model 1, Model 2 (measurement model) was defined with the path coefficients of these two groups being equal. The results showed that Model 1 fit the data well: χ^2^(14) = 120.763, CFI = 0.976, TLI = 0.927, and RMSEA = 0.081. Model 2 also fit the data well: χ^2^(23) = 140.741, CFI = 0.973, TLI = 0.951, and RMSEA = 0.067. The model comparison results showed that the differences between Model 1 and Model 2 were significant [Δχ^2^(9) = 19.978, *p* = 0.018]. Therefore, there were significant gender-based differences in the model in which PTSD mediated the association between pandemic exposure and conflict behaviors and online aggressive behaviors.

To investigate specific gender-based differences in the mediation model, we compared the path coefficients of the male and female models (see Table 2). The results showed that for male students, pandemic exposure directly and positively predicted conflict behaviors and online aggressive behaviors, but the predictive effect of pandemic exposure on PTSD symptom clusters was non-significant. Intrusive symptoms negatively predicted conflict behaviors and online aggressive behaviors, whereas hyperarousal symptoms positively predicted these two behaviors. For female students, pandemic exposure had non-significant direct relations with conflict behaviors and online aggressive behaviors, but significantly and positively predicted intrusive symptoms, hyperarousal symptoms, and NACM symptoms. Hyperarousal symptoms significantly and positively predicted conflict behaviors and online aggressive behaviors, whereas intrusive symptoms significantly and negatively predicted conflict behaviors. The relation between intrusive symptoms and online aggressive behaviors was non-significant. These results showed that in males, pandemic exposure directly and positively predicted conflict behaviors and online aggressive behaviors, but did not indirectly affect these two behaviors through PTSD symptom clusters. For females, pandemic exposure did not directly affect conflict and online aggressive behaviors, but predicted these two behaviors via PTSD symptom clusters. This means that pandemic exposure reduced female students’ conflict behaviors through inducing intrusive symptoms, and increased their conflict behaviors and online aggressive behaviors through inducing hyperarousal symptoms.

**TABLE 2:** Path coefficients in male and female students.

Paths	Male	Female
	β	β
Pandemic exposure → Conflict behaviors	0.139***	0.054
Pandemic exposure → Online aggressive behaviors	0.196***	0.050
Pandemic exposure → Intrusive symptom	0.008	0.097**
Pandemic exposure → NACM	0.002	0.087**
Pandemic exposure → Hyperarousal symptom	0.001	0.101***
Intrusive symptom → Conflict behaviors	−0.241***	−0.177**
Hyperarousal symptom → Conflict behaviors	0.531***	0.477***
Intrusive symptom → Online aggressive behaviors	−0.183**	–0.042
Hyperarousal symptom → Online aggressive behaviors	0.367***	0.176**

***p < 0.001, **p < 0.01 NACM, negative alterations in cognition and mood.

From the perspective of the whole model, the global prediction coefficient of female students’ pandemic exposure to their conflict behaviors was 0.085 (0.054 + 0.097 × (–0.177) + 0.101 × 0.477), and the global prediction coefficient to online aggressive behaviors was 0.064 (0.050 + 0.097 × (–0.042) + 0.101 × 0.176). This means that pandemic exposure could still induce conflict behaviors and online aggressive behaviors among female college students. In addition, we found that the global prediction coefficient for male students’ pandemic exposure to their conflict behavior was 0.138 (0.139 + 0.008 × (–0.241) + 0.001 × 0.531), and the global prediction coefficient to online aggressive behaviors was 0.195 (0.196 + 0.008 × (–0.183) + 0.001 × 0.367). These coefficients were significantly higher than the coefficients for females. Compared with females, male students’ pandemic exposure induced more conflict behaviors and online aggressive behaviors.

## Discussion

This study investigated the mechanism by which pandemic exposure influenced conflict behaviors and online aggressive behaviors through PTSD symptom clusters among college students. The results showed that college students’ pandemic exposure significantly and positively predicted their conflict behaviors and online aggressive behaviors, which was consistent with previous studies (12). Based on Dollard’s frustration-aggression hypothesis and Ben-Zur’s interpretation model about risk behaviors (27, 44), individuals may take aggressive behaviors as the attempt to repair, terminate, or avoid negative emotional states (45). Given the large amount of negative information from pandemic exposure, a variety of negative emotions emerged in individuals, which may lead to emotion dysregulation (46). Emotions are of great importance in those unfamiliar and complex situations (47), such as when exposed to COVID-19 or other public health emergencies. Aggressive behaviors may be regarded as a strategy to repair and manage these negative emotions. Individuals may hope to adjust their behavioral reactions and change their emotional experiences through conflict behaviors and online aggressive behaviors, thereby moderating the impacts of negative emotions.

In addition, we found that pandemic exposure negatively predicted conflict and online aggressive behaviors through the PTSD intrusive symptom cluster. The intrusive symptom cluster encompasses a series of uncontrolled and intrusive repeated experiences of traumatic events (29), and causes the individual to experience intense and lasting psychological pain. The COVID-19 pandemic had huge implications and lasted for a long time. Pandemic information from reality and cyber media may amplify individuals’ perception of risk (48), and individuals were repeatedly exposed to the pandemic; therefore, pandemic exposure significantly predicted their intrusive symptoms. The more serious the intrusive symptoms are, the more individuals may be immersed in negative ruminating on pandemic exposure (49), resulting in strong emotional experiences and memories. Individuals immersed themselves in negative emotions, and this may reduce their external behaviors. Therefore, pandemic exposure negatively predicted conflict behaviors and online aggressive behaviors through the intrusive symptom cluster.

Moreover, pandemic exposure positively predicted conflict and online aggressive behaviors through hyperarousal symptoms. The main symptoms of the hyperarousal cluster are excessive vigilance, easy to anger with little provocation, and possibly reckless behaviors (29). This study found that pandemic exposure significantly predicted hyperarousal symptoms. We assumed that with pandemic spread more and more widely, the diagnosed cases increased, and prevention and control measures progressed, people became more alert to face-to-face communication. With the increase of pandemic exposure, people’s alertness may also increase. This finding was consistent with previous studies (46, 50). Furthermore, in accordance with the cognitive action theory of PTSD (8, 26), individuals with hyperarousal symptoms may have cognitive biases when processing information. For example, they may be oversensitive and have intensified experiences of angry even facing a small amount of threatening information (51), resulting in strong aggressive behaviors. Therefore, pandemic exposure positively predicted conflict behaviors and online aggressive behaviors through the hyperarousal symptom cluster.

We found that pandemic exposure did not indirectly predict conflict behaviors and online aggressive behaviors through avoidance symptoms. One reason for this result may be that pandemic exposure could not significantly predict avoidance symptoms. The main manifestation of avoidance symptoms is the continuous avoidance of memories, thoughts, and external stimuli related to traumatic events. At the time of our investigation, the pandemic continues to rebound in China, but the momentum of pandemic prevention and control had been consolidated. People are willing to understand the relevant knowledge of pandemic and learn how to prevent COVID-19, especially for college students who have stronger adaptability and information processing ability. Therefore, the relation between pandemic exposure and avoidance symptoms was non-significant in this study. In addition, our study found that avoidance symptoms did not directly predict conflict behaviors and online aggressive behaviors. We assumed that individuals with high avoidance symptoms may be more likely to adopt cognitive avoidance and behavioral avoidance to deal with problems (52), rather than showing direct and explicit aggressive behaviors. Therefore, the indirect relation between pandemic exposure and these two behaviors through the avoidance symptom cluster was non-significant.

We also found that pandemic exposure did not predict conflict behaviors and online aggressive behaviors through NACM symptoms. Individuals with NACM symptoms mainly manifest as immersing themselves in negative emotions for a long time, having magnified negative beliefs and expectations about themselves, others and the world, and they may alienate others (29). This study found that pandemic exposure significantly predicted NACM symptoms. The transmission of COVID-19 is hidden, and pandemic prevention and control measures require people to enforce social distancing (53). Therefore, individuals may lack trust in others, and their strong negative emotions toward the outside world resulted in NACM symptoms. Individuals with NACM symptoms may have fewer positive emotions and may generalize negative emotions, resulting in negative self-cognition/emotions and significantly reduced participation and interest in external activities. Therefore, the correlation between NACM symptoms and explicit aggressive behaviors was non-significant, and pandemic exposure could not predict conflict behaviors and online aggressive behaviors through the NACM symptom cluster.

We showed that gender played a moderating role in the relations between pandemic exposure, PTSD symptom clusters, conflict behaviors, and online aggressive behaviors, which supported our hypothesis. Previous studies (54) found that after traumatic events, females showed more PTSD symptoms than males and males had higher rates of behavioral problems than females (32, 55). We assumed that this may be explained by the differences in coping strategies between males and females (56). During the pandemic, males might deal with pressure directly and explicitly, and they tended to adopt explicit behaviors to “fight” with problems. Thus, pandemic exposure directly predicted their conflict and online aggressive behaviors. Compared with males, females were more likely to use emotion-focused coping strategies (57) to tackle the stress following the pandemic, resulting in their intrusive and hyperarousal symptoms. When intrusive symptoms appear, females may not be able to deal with their problems alone and therefore seek more social support than usual (58). However, the decrease in interpersonal communication during the pandemic meant that females could access less social support, and therefore immersed themselves in mental pain and reduced external aggressive behaviors. When hyperarousal symptoms appear, females may become extremely sensitive to external information, easy to anger, and show more aggressive behaviors. Pandemic exposure indirectly affected conflict and online aggressive behaviors mainly through these two symptoms. Therefore, we proposed that gender played a moderating role in the relations between pandemic exposure, PTSD symptom clusters, conflict behaviors, and online aggressive behaviors.

The findings indicate that during the COVID-19 pandemic or other future public health emergencies, health care departments should not only pay attention to the youth’s psychological symptoms, but also care about their behavioral problems to avoid potential public unrest, since pandemic exposure can trigger conflict behaviors and online aggressive behaviors via PTSD symptoms. What is more, gender differences should be considered while carrying out psychological intervention. Priority can be given to targeted intervention on the risk of males’ behavioral problems and females’ PTSD symptoms.

Several limitations of the present study should be noted. First, different people in different pandemic phrases may have distinct psychological and behavioral responses to the pandemic, thus the generalization of our findings to other populations in other public health events should be with caution. Second, the cross-sectional design of this study did not indicate the causal relations between the variables. Third, for safety reasons, we carried out this study by using online questionnaires, future research can consider additional interviews or experimental designs to deepen the findings.

## Conclusion

The present study found that intrusive symptoms and hyperarousal symptoms mediated the relations between pandemic exposure and conflict behaviors and online aggressive behaviors in college students, and gender played a moderating role in the above mediation model. Our findings enriched body of knowledge regarding the underlying mechanism that pandemic exposure influencing conflict behaviors and online aggressive behaviors in male and female populations, which helped to inform the mental health service system in a public health emergency.

## Data Availability Statement

The raw data supporting the conclusions of this article will be made available by the authors, without undue reservation.

## Ethics Statement

The studies involving human participants were reviewed and approved by the Research Ethics Committee of the Department of Psychology and Behavioral Sciences, Zhejiang University. The patients/participants provided their written informed consent to participate in this study.

## Author Contributions

BZ contributed to the design of the study, analysis of data, and writing and revision of the manuscript. BY contributed to the visualization, project administration, and writing and revision of the manuscript. XZ contributed to the collection of data, analysis of data, writing and revision of the manuscript. All authors contributed to the article and approved the submitted version.

## Conflict of Interest

The authors declare that the research was conducted in the absence of any commercial or financial relationships that could be construed as a potential conflict of interest.

## Publisher’s Note

All claims expressed in this article are solely those of the authors and do not necessarily represent those of their affiliated organizations, or those of the publisher, the editors and the reviewers. Any product that may be evaluated in this article, or claim that may be made by its manufacturer, is not guaranteed or endorsed by the publisher.
